# Development and validation of a machine learning-based risk prediction model for stroke-associated pneumonia in older adult hemorrhagic stroke

**DOI:** 10.3389/fneur.2025.1591570

**Published:** 2025-06-18

**Authors:** Yi Cao, Haipeng Deng, Shaoyun Liu, Xi Zeng, Yangyang Gou, Weiting Zhang, Yixinyuan Li, Hua Yang, Min Peng

**Affiliations:** ^1^Department of Neurosurgery, Affiliated Hospital of Guizhou Medical University, Guiyang, China; ^2^School of Nursing, Guizhou Medical University, Guiyang, China; ^3^Department of Nursing Quality Management, Affiliated Hospital of Guizhou Medical University, Guiyang, China

**Keywords:** machine learning, older adult, hemorrhagic stroke, stroke-associated pneumonia, prediction model, validation

## Abstract

**Objective:**

To develop and validate a machine learning (ML)-based model for predicting stroke-associated pneumonia (SAP) risk in older adult hemorrhagic stroke patients.

**Methods:**

A retrospective collection of older adult hemorrhagic stroke patients from three tertiary hospitals in Guiyang, Guizhou Province (January 2019–December 2022) formed the modeling cohort, randomly split into training and internal validation sets (7:3 ratio). External validation utilized retrospective data from January–December 2023. After univariate and multivariate regression analyses, four ML models (Logistic Regression, XGBoost, Naive Bayes, and SVM) were constructed. Receiver operating characteristic (ROC) curves and area under the curve (AUC) were calculated for training and internal validation sets. Model performance was compared using Delong's test or Bootstrap test, while sensitivity, specificity, accuracy, precision, recall, and F1-score evaluated predictive efficacy. Calibration curves assessed model calibration. The optimal model underwent external validation using ROC and calibration curves.

**Results:**

A total of 788 older adult hemorrhagic stroke patients were enrolled, divided into a training set (*n* = 462), an internal validation set (*n* = 196), and an external validation set (*n* = 130). The incidence of SAP in older adult patients with hemorrhagic stroke was 46.7% (368/788). Advanced age [OR = 1.064, 95% CI (1.024, 1.104)], smoking[OR = 2.488, 95% CI (1.460, 4.24)], low GCS score [OR = 0.675, 95% CI (0.553, 0.825)], low Braden score [OR = 0.741, 95% CI (0.640, 0.858)], and nasogastric tube [OR = 1.761, 95% CI (1.048, 2.960)] were identified as risk factors for SAP. Among the four machine learning algorithms evaluated [XGBoost, Logistic Regression (LR), Support Vector Machine (SVM), and Naive Bayes], the LR model demonstrated robust and consistent performance in predicting SAP among older adult patients with hemorrhagic stroke across multiple evaluation metrics. Furthermore, the model exhibited stable generalizability within the external validation cohort. Based on these findings, the LR framework was subsequently selected for external validation, accompanied by a nomogram visualization. The model achieved AUC values of 0.883 (training), 0.855 (internal validation), and 0.882 (external validation). The Hosmer-Lemeshow (H-L) test indicates that the calibration of the model is satisfactory in all three datasets, with *P*-values of 0.381, 0.142, and 0.066 respectively.

**Conclusions:**

This study constructed and validated a risk prediction model for SAP in older adult patients with hemorrhagic stroke based on multi-center data. The results indicated that among the four machine learning algorithms (XGBoost, LR, SVM, and Naive Bayes), the LR model demonstrated the best and most stable predictive performance. Age, smoking, low GCS score, low Braden score, and nasogastric tube were identified as predictive factors for SAP in these patients. These indicators are easily obtainable in clinical practice and facilitate rapid bedside assessment. Through internal and external validation, the model was proven to have good generalization ability, and a nomogram was ultimately drawn to provide an objective and operational risk assessment tool for clinical nursing practice. It helps in the early identification of high-risk patients and guides targeted interventions, thereby reducing the incidence of SAP and improving patient prognosis.

## 1 Introduction

Stroke-associated pneumonia (SAP) refers to newly acquired pneumonia in non-mechanically ventilated patients within 7 days of stroke onset (1). First proposed by German scholar Hilker in 2003 (2), subsequent studies report its incidence rate ranging from 6.5 to 58.4%, with risk factors including advanced age, male sex, smoking, dysphagia, hyperglycemia, and lower Glasgow Coma Scale (GCS) scores (3–8). Compared to non-SAP patients, SAP significantly worsens prognosis, leading to increased disability and mortality rates, prolonged hospitalization, and elevated healthcare costs (3, 5, 8–10). Meanwhile, with the intensification of population aging in China, the nursing needs of older adult patients with hemorrhagic stroke are becoming increasingly prominent (11). Current nursing strategies have deficiencies in aspects such as infection prevention, individualized intervention, and uneven distribution of medical resources. Especially in the context of limited medical resources, there is a lack of validated tools to prioritize the identification of high-risk patients and optimize nursing priorities, which restricts the prevention and control of SAP.

Machine Learning (ML) a subset of artificial intelligence (AI), enables in-depth exploration and analysis of extensive datasets, offering novel methodologies and research frameworks for precise prediction. Its applications span diverse fields, particularly in medicine, where ML facilitates the development of automated tools for clinical decision-making based on multidimensional medical data (10, 12). Risk prediction models, initially applied in cardiothoracic surgery (13, 14), leverage patient-specific risk factors and ML algorithms to forecast disease progression, therapeutic responses, and outcomes. Recent studies have utilized ML to integrate vital signs, epidemiological data, and laboratory/imaging findings for diagnostic or prognostic purposes. However, there are currently few risk prediction models for SAP in older adult patients with hemorrhagic stroke based on ML algorithms. The absence of such models not only limits the clinical early-warning ability but also hinders the precise allocation of nursing resources. This need is particularly urgent considering the characteristics of older adult patients with multiple underlying diseases and a short window period for nursing intervention. In this study, the prediction of SAP in older adult patients with hemorrhagic stroke was defined as a binary classification problem. Therefore, four widely used ML algorithms for solving classification problems (Logistic regression, Naive Bayes, Support Vector Machine, and eXtreme Gradient Boosting algorithm) were selected to construct the risk prediction model. The effectiveness of the model was evaluated through internal and external validation, aiming to provide references for clinical nursing practice, prevention, and control.

## 2 Materials and methods

### 2.1 Study population

We retrospectively collected data from older adult patients with hemorrhagic stroke who were admitted to three tertiary hospitals in Guiyang City, Guizhou Province, between January 2019 and December 2022. These patients served as the modeling group and were randomly divided into training and internal validation sets according to a 7:3 ratio. Additionally, we retrospectively gathered clinical data from older adult hemorrhagic stroke patients admitted to the same hospitals between January 2023 and December 2023 for external validation purposes. Inclusion Criteria: (1) Patients aged ≥60 years. (2) Patients diagnosed with hemorrhagic stroke based on International Classification of Diseases, 10th Revision (ICD-10) codes I60-I61 (subarachnoid hemorrhage-intracerebral hemorrhage) (15). (3) Time from onset of symptoms to hospital admission ≤ 48 hours. Exclusion Criteria: (1) Patients with severe pre-existing cardiac, renal, or hepatic dysfunction. (2) Patients with a history of mental illness. (3) Patients with incomplete core clinical data. (4) Patients already diagnosed with other pulmonary infectious diseases prior to admission.

### 2.2 Research methods

#### 2.2.1 Sample size calculation

The empirical rule for Logistic regression is that the sample size should be 10–15 times the number of independent variables to ensure the robustness of the results (16). Moreover, the prevalence of stroke-related pneumonia in older adult patients with hemorrhagic stroke was found to be 63.5%. There were a total of 5 predictive influencing variables in this study. The sample size for the modeling was *N* = 5 × 15 ÷ (1–63.5%) ≈ 205 cases, with a ratio of 7:3 for the training set and validation set. The sample size of the training set was *N* × 7/10 ≈ 144 cases, and the sample size of the validation set was *N* × 3/10 ≈ 61 cases. The external validation sample size was ≥ 100 cases (17). This study required a minimum sample size of 144 + 61 + 100 = 305 cases.

#### 2.2.2 General information

All patient data are sourced from the hospital's electronic medical record system. The data collected included sex, age, treatment costs, type of hemorrhagic stroke, length of stay (LOS), educational level, ethnicity, smoking, drinking, hypertension, diabetes, Glasgow Coma Scale (GCS) score, Modified Rankin Scale (mRS), Braden Scale, systolic blood pressure (SBP), diastolic blood pressure (DBP), Body Mass Index (BMI), white blood cell count (WBC), neutrophil count (Neut), lymphocyte count (Lym), monocyte count (Mono), red blood cell count (RBC), platelet count (PLT), hematocrit (HCT), activated partial thromboplastin time (APTT), blood glucose (Glu), nasogastric tube, and use of acid suppressants.

#### 2.2.3 Diagnostic criteria for SAP

The modified criteria of the Centers for Disease Control and Prevention (CDC) were used as the diagnostic standard for stroke-associated pneumonia (SAP) (18). Two physicians independently assessed the chest X-ray or CT scan results of patients and diagnosed SAP based on these criteria. In cases of diagnostic disagreement, a senior physician was consulted until a consensus was reached.

### 2.3 Statistical analysis

Statistical analysis of the data was performed using R 4.2.3 software. The core-related data with missing values < 20% in the study (such as Educational level, mRS score) were filled using the multiple imputation method and then randomly split at a ratio of 7:3. Based on the data distribution, measurement data following a normal distribution were presented as mean ± standard deviation, and the independent-samples *t*-test was used for comparisons between two groups. For measurement data with a skewed distribution, they were presented as median and inter-quartile range [*M*(*Q1, Q2*)], and the non-parametric Mann–Whitney U rank-sum test was used for group comparisons. Count data were presented as frequency (*n*) and percentage (%), and the chi-square test was used for group comparisons. The significance level was set as two-sided α = 0.05. Variables with *P* < 0.05 in the univariate analysis and those considered clinically meaningful were included in the multivariate regression analysis. Collinearity analysis was performed for each variable. Tolerance was classified into severe collinearity (Tolerance < 0.1), moderate collinearity (0.1 ≤ Tolerance < 0.2), and no collinearity (Tolerance ≥ 0.2). The variance inflation factor (VIF) was divided into severe collinearity (VIF > 10), moderate collinearity (5 < VIF ≤ 10), and no collinearity (VIF ≤ 5). Four machine-learning models were constructed: XGBoost, Naive Bayes, Support Vector Machine (SVM), and Logistic Regression (LR). ROC curves were plotted, and AUC values were calculated in both training and validation sets, with models categorized as excellent (AUC ≥ 0.9), good (0.7 ≤ AUC < 0.9), fair (0.5 ≤ AUC < 0.7), or poor (AUC < 0.5). DeLong's test or Bootstrap test was employed to compare the AUCs of the models pairwise. The predictive performance of the models was evaluated using sensitivity, specificity, accuracy, precision, recall, and F1-score. The calibration of the models was assessed using calibration curves, and the Hosmer–Lemeshow (H–L) test was used to evaluate the goodness-of-fit of the models. After selecting the optimal model, in the external validation, ROC curves and calibration curves were plotted to evaluate the discrimination and calibration of the risk prediction model. The model was visualized by plotting a nomogram. Finally, the clinical utility of the model was evaluated through Decision Curve Analysis (DCA). All analyses were conducted using the following R packages: caret, mice, Xgboost, naivebayes, e1071, MASS, pROC, dplyr, rms, Hmisc, rmda, etc.

## 3 Results

### 3.1 General characteristics

**Figure 1:**
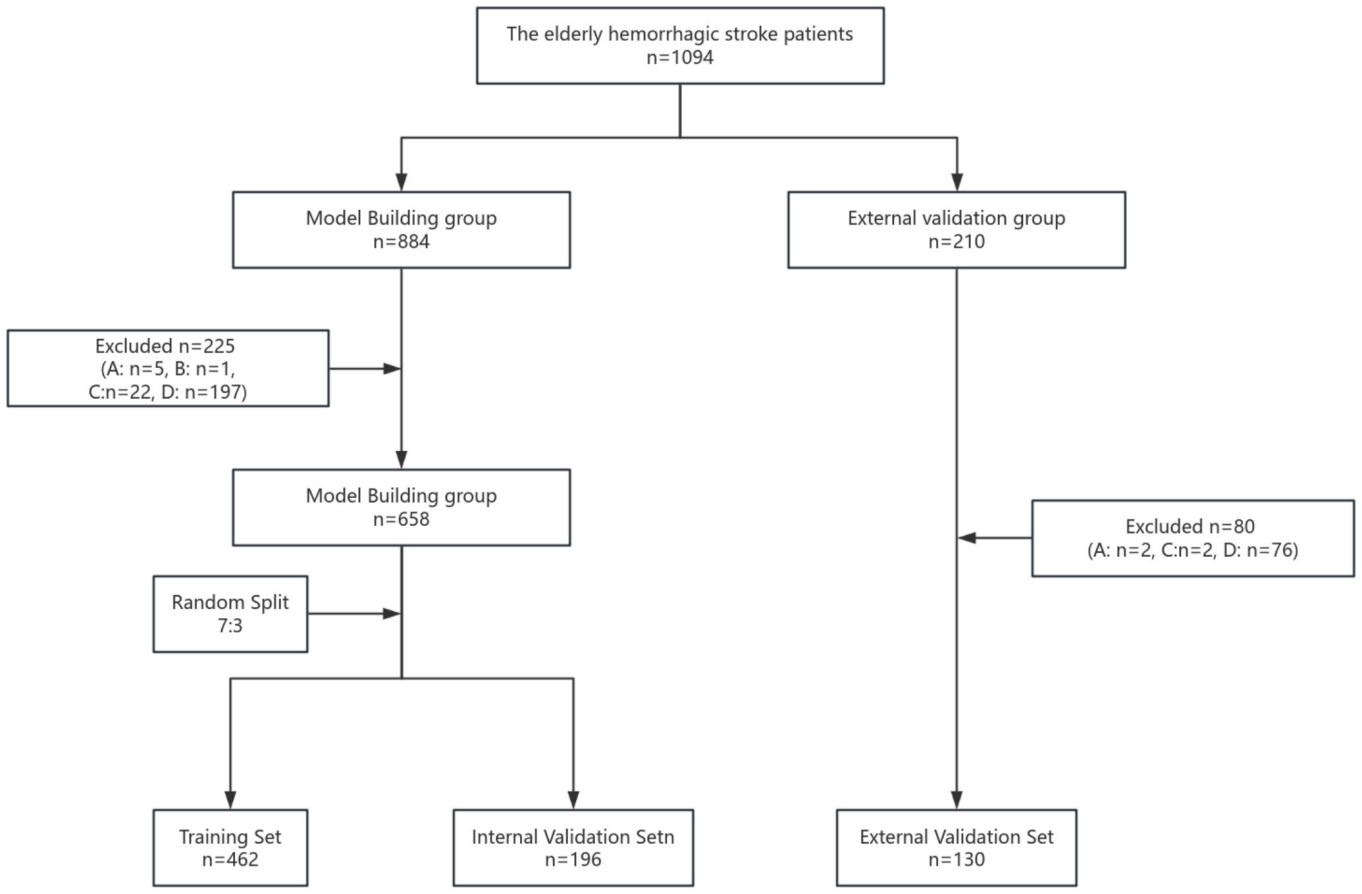
Flowchart of patient inclusion for older adult patients with hemorrhagic stroke. **(A)** Patients with severe pre-existing cardiac, renal, or hepatic dysfunction before admission; **(B)** patients with a history of mental illness; **(C)** patients with incomplete clinical core data; **(D)** patients with pre-admission diagnosis of other pulmonary infectious diseases.

In this study, 658 older adult patients with hemorrhagic stroke were included for model construction. After random allocation using a 7:3 ratio, the training set included 462 patients, and the internal validation set included 196 patients. Additionally, 130 older adult patients with hemorrhagic stroke were enrolled for external validation, as shown in Figure 1. In the training set, internal validation set and external validation set, there was no significant difference in each variable among the three data sets (*P* ≥ 0.05) in Table 1.

**Table 1:** Comparison of general characteristics among the training set, internal validation set, and external validation set.

**Variables**	**Training set (*n* = 462)**	**Internal validation set (*n* = 196)**	**External validation set (*n* = 130)**	**χ^2^/H value**	***P*-value**
**SAP [cases (%)]**				0.364	0.834
No	248 (53.7%)	101 (51.5%)	71 (54.6%)		
Yes	214 (46.3%)	95 (48.5%)	59 (45.4%)		
Age (years)	69.4 ± 7.37	69.6 ± 7.74	69.7 ± 7.30	0.420	0.834
**Sex [cases (%)]**				0.406	0.816
Female	164 (35.5%)	73 (37.2%)	44 (33.8%)		
Male	298 (64.5%)	123 (62.8%)	86 (66.2%)		
**Type of hemorrhagic stroke [cases (%)]**				0.295	0.863
Subarachnoid hemorrhage	73 (15.8%)	28 (14.3%)	21 (16.2%)		
Intracerebral hemorrhage	389 (84.2%)	168 (85.7%)	109 (83.8%)		
**Educational level [cases (%)]**				1.210	0.976
College or above	40 (8.66%)	14 (7.14%)	9 (6.92%)		
High school or vocational school	65 (14.1%)	31 (15.8%)	18 (13.8%)		
Junior high school	132 (28.6%)	59 (30.1%)	39 (30.0%)		
Elementary school or below	225 (48.7%)	92 (46.9%)	64 (49.2%)		
**Ethnicity [cases (%)]**				0.284	0.868
Han nationality	406 (87.9%)	171 (87.2%)	112 (86.2%)		
Others	56 (12.1%)	25 (12.8%)	18 (13.8%)		
**Smoking [cases (%)]**				1.399	0.497
No	263 (56.9%)	121 (61.7%)	74 (56.9%)		
Yes	199 (43.1%)	75 (38.3%)	56 (43.1%)		
**Drinking [cases (%)]**				1.924	0.382
No	298 (64.5%)	132 (67.3%)	92 (70.8%)		
Yes	164 (35.5%)	64 (32.7%)	38 (29.2%)		
**Hypertension [cases (%)]**				0.467	0.792
No	144 (31.2%)	65 (33.2%)	44 (33.8%)		
Yes	318 (68.8%)	131 (66.8%)	86 (66.2%)		
**Diabetes [cases (%)]**				3.571	0.168
No	417 (90.3%)	167 (85.2%)	116 (89.2%)		
Yes	45 (9.74%)	29 (14.8%)	14 (10.8%)		
GCS (points)	13.4 ± 2.80	13.1 ± 2.90	13.2 ± 3.06	2.134	0.344
mRS (points)	3.43 ± 1.16	3.58 ± 1.15	3.42 ± 1.10	4.006	0.135
Braden (points)	14.3 ± 2.78	14.1 ± 3.12	14.5 ± 2.97	3.209	0.200
SBP (mmHg)	162 ± 27.8	161 ± 28.7	160 ± 27.6	0.894	0.640
DBP (mmHg)	93.1 ± 16.6	92.3 ± 15.8	91.8 ± 15.0	0.296	0.862
BMI (kg/m^2^)	23.4 ± 3.40	24.0 ± 3.81	23.6 ± 3.57	3.053	0.217
WBC (10^9^/L)	7.47 ± 1.67	7.34 ± 1.75	7.43 ± 1.84	0.888	0.641
Neut (10^9^/L)	5.79 ± 2.07	5.83 ± 2.18	5.70 ± 2.14	0.598	0.742
Lym (10^9^/L)	1.40 ± 0.73	1.33 ± 0.67	1.43 ± 0.66	1.769	0.413
Mono (10^9^/L)	0.45 ± 0.18	0.43 ± 0.19	0.46 ± 0.18	3.004	0.223
RBC (10^12^/L)	4.57 ± 0.58	4.56 ± 0.60	4.63 ± 0.58	1.853	0.396
PLT (10^9^/L)	188 ± 53.0	188 ± 57.5	193 ± 58.3	0.545	0.762
HCT (%)	42.0 ± 4.46	41.9 ± 4.86	42.2 ± 4.74	0.813	0.666
APTT (s)	33.3 ± 5.07	32.9 ± 6.42	33.4 ± 4.90	2.740	0.254
Glu (mmol/L)	7.13 ± 2.46	7.38 ± 2.55	7.17 ± 2.46	1.593	0.451
**Nasogastric tube [cases (%)]**				0.567	0.972
No	229 (49.6%)	96 (49.0%)	63 (48.5%)		
Yes	233 (50.4%)	100 (51.0%)	67 (51.5%)		
**Use of acid suppressants [cases (%)]**				3.370	0.185
No	362 (78.4%)	142 (72.4%)	95 (73.1%)		
Yes	100 (21.6%)	54 (27.6%)	35 (26.9%)		

### 3.2 Univariate analysis of the training set

Univariate analysis revealed statistically significant differences between SAP patients and no-SAP patients in the training set for age, smoking, GCS, mRS, Braden scale, SBP, DBP, WBC, Neut, Lym, Glu, nasogastric tube, and use of acid suppressants (*P* < 0.05). The results are presented in Table 2.

**Table 2:** Univariate analysis of the training set.

**Variables**	**No-SAP (*n* = 248)**	**SAP (*n* = 214)**	***χ^2^*/*t* Value**	***P*-value**
Age (years)	68.2 ± 6.7	70.7 ± 7.8	−3.682	**< 0.001**
**Sex [cases (%)]**			3.406	0.065
Female	98 (39.5%)	66 (30.8%)		
Male	150 (60.5%)	148 (69.2%)		
**Type of hemorrhagic stroke [cases (%)]**			1.437	0.231
Subarachnoid hemorrhage	34 (13.7%)	39 (18.2%)		
Intracerebral hemorrhage	214 (86.3%)	175 (81.8%)		
**Educational level [cases (%)]**			3.697	0.296
College or above	24 (9.7%)	16 (7.5%)		
High school or vocational school	39 (15.7%)	26 (12.1%)		
Junior high school	74 (29.8%)	58 (27.1%)		
Elementary school or below	111 (44.8%)	114 (53.3%)		
**Ethnicity [cases (%)]**			2.418	0.120
Han nationality	36 (14.5%)	20 (9.3%)		
Others	212 (85.5%)	194 (90.7%)		
**Smoking [cases (%)]**			13.255	**< 0.001**
No	161 (64.9%)	102 (47.7%)		
Yes	87 (35.1%)	112 (52.3%)		
**Drinking [cases (%)]**			3.456	0.063
No	170 (68.5%)	128 (59.8%)		
Yes	78 (31.5%)	86 (40.2%)		
**Hypertension [cases (%)]**			0.716	0.397
No	82 (33.1%)	62 (29%)		
Yes	166 (66.9%)	152 (71%)		
**Diabetes [cases (%)]**			1.869	0.172
No	219 (88.3%)	198 (92.5%)		
Yes	29 (11.7%)	16 (7.5%)		
GCS (points)	14.6 ± 1.0	11.9 ± 3.4	11.134	**< 0.001**
mRS (points)	2.9 ± 1.2	4.0 ± 0.8	−11.431	**< 0.001**
Braden (points)	15.7 ± 2.7	12.7 ± 1.8	13.711	**< 0.001**
SBP (mmHg)	156.1 ± 25.3	168.1 ± 29.3	−4.699	**< 0.001**
DBP (mmHg)	91.1 ± 16.2	95.4 ± 16.8	−2.770	**0.006**
BMI (kg/m^2^)	23.6 ± 3.3	23.3 ± 3.5	0.833	0.403
WBC (10^9^/L)	7.3 ± 1.8	7.7 ± 1.5	−3.075	**0.002**
Neut (10^9^/L)	5.4 ± 2.0	6.3 ± 2.1	−4.826	**< 0.001**
Lym (10^9^/L)	1.5 ± 0.6	1.3 ± 0.8	2.109	**0.036**
Mono (10^9^/L)	0.4 ± 0.2	0.5 ± 0.2	−0.444	0.656
RBC (10^12^/L)	4.6 ± 0.6	4.6 ± 0.6	0.496	0.619
PLT (10^9^/L)	188.1 ± 50.7	188.7 ± 55.7	−0.117	0.906
HCT (%)	42.1 ± 4.0	41.9 ± 5.0	0.607	0.544
APTT (s)	33.3 ± 4.8	33.3 ± 5.4	0.051	0.959
Glu (mmol/L)	6.8 ± 2.3	7.5 ± 2.6	−2.996	**0.003**
**Nasogastric tube [cases (%)]**			14.74	**< 0.001**
No	144 (58.1%)	85 (39.7%)		
Yes	104 (41.9%)	129 (60.3%)		
**Use of acid suppressants [cases (%)]**			91.318	**< 0.001**
No	237 (95.6%)	125 (58.4%)		
Yes	11 (4.4%)	89 (41.6%)		

The bold values provided: Variables with *P* < 0.05.

### 3.3 Multivariate logistic regression analysis of the training set

Collinearity analysis was conducted on the variables with *P* < 0.05 in the univariate analysis and those deemed clinically significant through discussions within the research group. The results revealed that only WBC and Neut showed moderate collinearity, while the remaining variables exhibited no collinearity (see Supplementary Table 1). The results of the multivariate regression analysis indicated that age, smoking, GCS score, Braden score, and Nasogastric tube were influencing factors for the occurrence of SAP in older adult patients with hemorrhagic stroke, as presented in Table 3. Although WBC and Neut demonstrated moderate collinearity, their inclusion did not affect the significance of the primary variables. Moreover, neither of them was statistically significant in the final model. We retained them to ensure the completeness of the analysis.

**Table 3:** Multivariate logistic regression analysis of the training set.

**Variables**	**Coefficient**	**Standard error**	**Z value**	**OR (95%CI)**	***P*-value**
Age	0.062	0.019	3.213	1.064 (1.024, 1.104)	**0.001**
Smoking	0.911	0.272	3.351	2.488 (1.460, 4.24)	**0.001**
GSC	−0.393	0.102	−3.850	0.675 (0.553, 0.825)	**< 0.001**
mRs	0.222	0.152	1.453	1.248 (0.926, 1.683)	0.146
Braden	−0.300	0.075	−4.017	0.741 (0.640, 0.858)	**< 0.001**
SBP	0.004	0.007	0.495	1.004 (0.989, 1.018)	0.620
DBP	0.006	0.012	0.461	1.006 (0.982, 1.030)	0.645
WBC	−0.151	0.181	−0.835	0.860 (0.604, 1.225)	0.404
Neut	0.188	0.161	1.165	1.206 (0.880, 1.654)	0.244
Lym	0.018	0.250	0.074	1.019 (0.624, 1.662)	0.941
Glu	0.050	0.054	0.919	1.051 (0.945, 1.168)	0.358
Use of acid suppressants	0.784	0.421	1.865	2.191 (0.961, 4.995)	0.062
Nasogastric tube	0.566	0.265	2.137	1.761 (1.048, 2.960)	**0.033**

The bold values provided: Variables with *P* < 0.05.

### 3.4 Comparison of model performance between the training set and internal validation set

**Figure 2:**
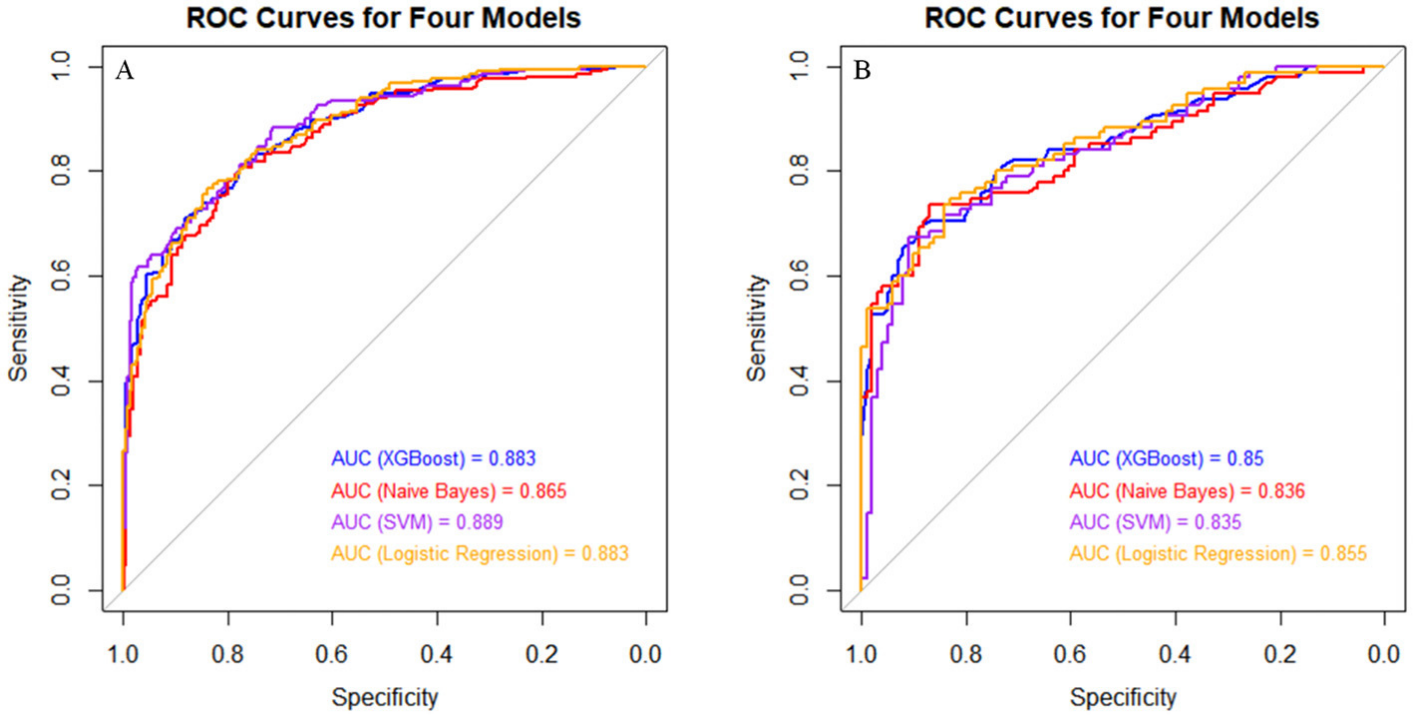
ROC curves of the four models for identifying SAP in the training set and the internal validation set. **(A)** Four models identify the ROC curve of SAP in the training set; **(B)** four models identify the ROC curve of SAP in the internal validation set.

Four machine learning (ML) models (XGBoost, SVM, Naive Bayes, and LR) were developed using the variables identified in the multivariate logistic regression analysis. The performance of the models, including AUC, sensitivity, specificity, accuracy, precision, recall, and F1 score, was evaluated in both the training set and internal validation set (see Supplementary Tables 2 and 3; see Figure 2). Pairwise comparisons of AUC values were also conducted (see Table 4).

**Table 4:** Pairwise comparisons of AUC values between the training set and internal validation set.

**Model comparison**	**Data set**	**Comparison method**	***D* value/*Z* value**	***P*-value**
XGBoost vs. Naive Bayes	Training	DeLong's test	2.765^a^	**0.006**
	Internal validation	DeLong's test	1.829^a^	0.067
XGBoost vs. SVM	Training	Bootstrap test	−0.306^b^	0.760
	Internal validation	Bootstrap test	0.375^b^	0.708
XGBoost vs. logistic regression	Training	DeLong's test	0.008^a^	0.993
	Internal validation	DeLong's test	−0.414^a^	0.679
Naive Bayes vs. SVM	Training	Bootstrap test	1.057^b^	0.290
	Internal validation	Bootstrap test	0.031^b^	0.976
Naive Bayes vs. logistic regression	Training	DeLong's test	2.495^a^	**0.013**
	Internal validation	DeLong's test	−1.824^a^	0.068
SVM vs. logistic regression	Training	Bootstrap test	0.297^b^	0.766
	Internal validation	Bootstrap test	−0.494^b^	0.612

^a^*Z* value; ^b^*D* value. The bold values provided: Variables with *P* < 0.05.

The results showed that XGBoost demonstrated excellent performance in the training set, with an AUC of 0.883 (0.853–0.913) and high specificity of 0.883 (0.843–0.923). However, its specificity decreased significantly in the internal validation set (0.695, 0.602–0.787). The LR model exhibited relatively stable performance across both the training and internal validation sets, with comparable AUC values (0.883 in the training set and 0.855 in the internal validation set) and high sensitivity (0.766 in the training set and 0.791 in the internal validation set). Specificity slightly decreased from 0.839 in the training set to 0.747 in the internal validation set. SVM showed the best sensitivity in the training set (0.883, 0.840–0.926) and the highest AUC (0.889, 0.860–0.918). However, its performance declined in the internal validation set, with an AUC of 0.835 (0.779–0.892), sensitivity of 0.796 (0.733–0.850), and the lowest specificity of 0.674 (0.579–0.768). Naive Bayes demonstrated consistent sensitivity in both the training set (0.808, 0.756–0.861) and internal validation set (0.806, 0.744–0.859) but performed poorly in terms of AUC.

Pairwise comparisons of AUC values revealed significant differences between XGBoost and Naive Bayes in the training set (*Z* = 2.765, *P* = 0.006) and between Naive Bayes and LR in the training set (*Z* = 2.495, *P* = 0.013). No significant differences were observed between XGBoost and SVM, XGBoost and LR, Naive Bayes and SVM, or SVM and LR in either the training set or internal validation set (*P* > 0.05). Based on the performance and stability of the models, the LR model has relatively stable and excellent performance in all aspects, especially in the internal validation set. Finally, the LR model is selected for external validation.

### 3.5 Model external validation

#### 3.5.1 Discrimination

**Figure 3:**
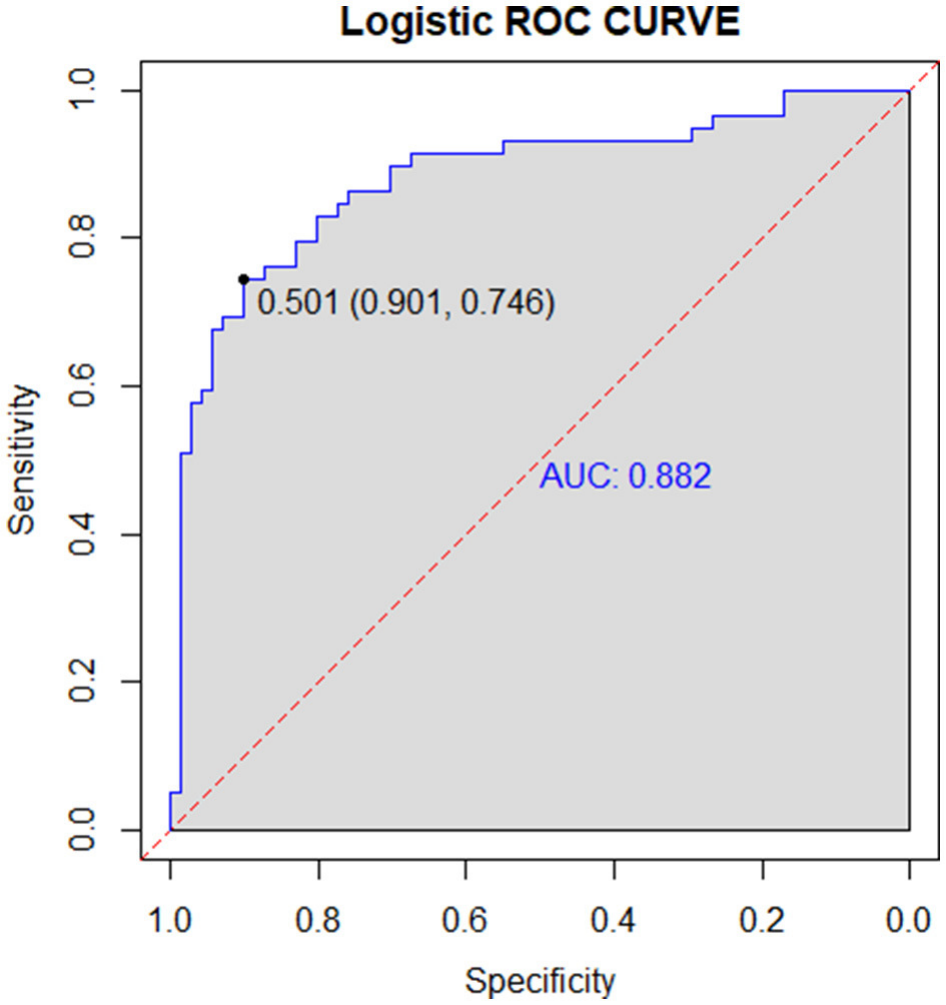
ROC curve for external validation.

The logistic regression model, which demonstrated superior and stable performance during internal validation, was subjected to external validation to assess its generalization and reliability across different datasets. The results showed an AUC of 0.882 (95% CI: 0.853–0.912) in the external validation set, as illustrated in Figure 3.

#### 3.5.2 Calibration

**Figure 4:**
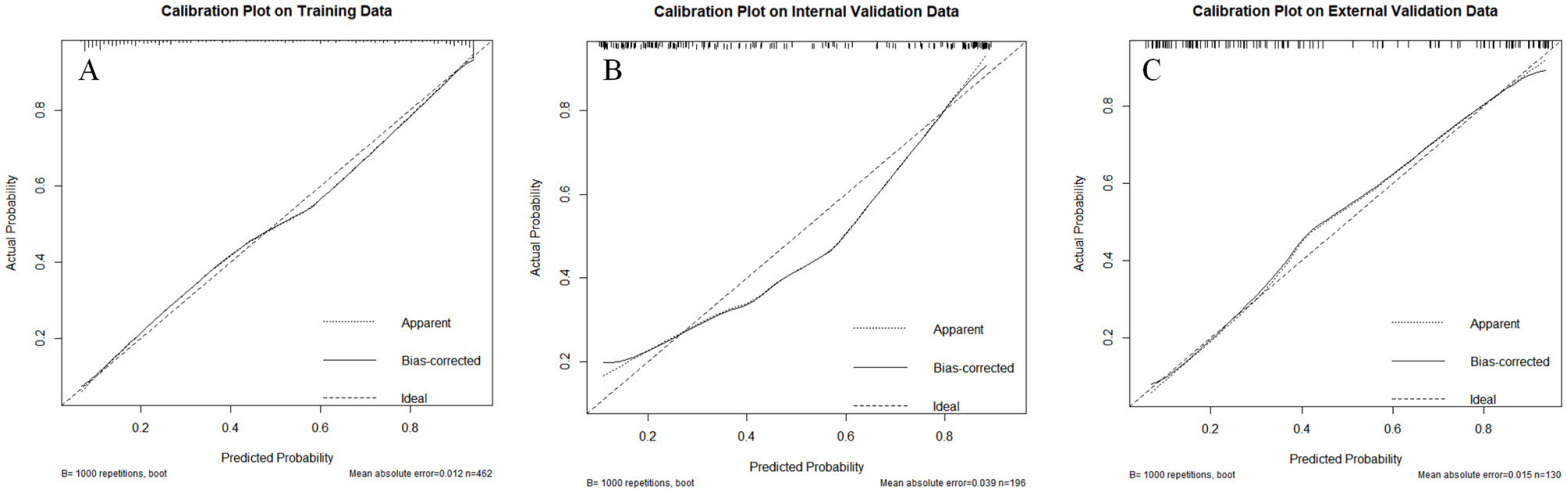
Calibration curves of the model in the training, internal validation and external validation. **(A)** Calibration plot of the training; **(B)** calibration plot of internal validation; **(C)** calibration plot in the external validation.

The calibration curves, based on the H-L test shows that the model has a good fit in the training set, internal validation, and external validation, with *P* values of 0.381, 0.142, and 0.066, respectively, indicating that the model has good calibration, as shown in Figure 4.

### 3.6 Model equation and nomogram

**Figure 5:**
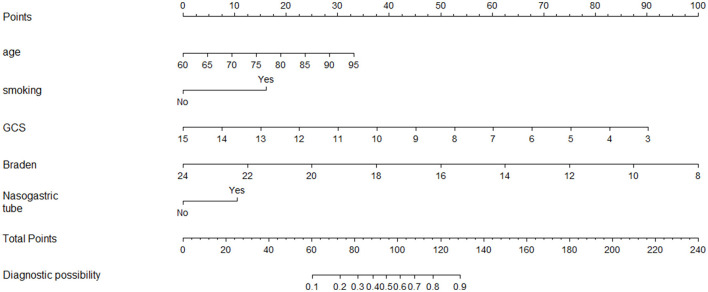
Nomogram for predicting stroke-associated pneumonia in older adult patients with hemorrhagic stroke.

The LR model equation is Logit(p) = 2.177 + 0.062 × (Age) + 0.911 × (Smoking) – 0.393 × (GCS) – 0.300 × (Braden) + 0.566 × (Nasogastric tube). A nomogram was plotted, as shown in Figure 5.

### 3.7 Clinical effectiveness

The decision curve analysis of the model is shown in Supplementary Figure 1, indicating a favorable clinical net benefit.

## 4 DIiscussion

### 4.1 Risk prediction model for SAP in older adult patients with hemorrhagic stroke

SAP is one of the most common respiratory complications in patients with hemorrhagic stroke, closely associated with adverse outcomes and increased mortality risk (6, 9, 15). In this study, the incidence of SAP among older adult patients with hemorrhagic stroke was 46.7% (368/788), potentially due to the rapid onset, swift hematoma expansion, and rapid progression of hemorrhagic stroke, often accompanied by consciousness disorders, dysphagia, and reduced cough reflex (3, 9, 19, 20). In addition, the patients in this study were older, and the medical environment in Guizhou Province, China, is still continuously improving overall compared to developed regions. This may partially explain the high incidence phenomenon. At the same time, with the development of data science and AI, data-driven methods using ML can greatly improve the development of medical prediction models, such as protein function design (21), personalized drug dosing (22), Parkinson's disease diagnosis and classification (23), etc. Therefore, to enhance the accuracy of early identification of high-risk individuals for SAP in older adult patients with hemorrhagic stroke, this study developed a risk prediction model.

During the process of building the predictive model, the results of four machine learning methods were compared. Each method has its own unique characteristics. Among them, the LR model showed excellent and stable predictive performance across different datasets. During the internal and external validation of the model, there was no sign of overfitting, demonstrating its robustness. Further consideration of the model's characteristics revealed that the LR model has significant advantages in simplicity and interpretability. When dealing with binary classification tasks, its principle and calculation process are relatively clear and understandable, making it convenient to provide reasonable explanations for the research results. In contrast, although XGBoost has certain advantages in handling complex data and improving model performance, Naive Bayes has unique applications in handling text classification tasks, and SVM performs well in handling high-dimensional data and non-linear classification problems. However, considering the specific requirements of this study and the characteristics of the data, the LR model, due to its simplicity, stability, and outstanding performance in binary classification tasks, becomes the most suitable model for this study. This finding aligns with previous research by Niu et al. (24), who compared 22 ML methods in alcoholic liver disease and ultimately selected the LR model.

### 4.2 Influencing factors and nursing interventions for SAP in older adult patients with hemorrhagic stroke

In this study, age, smoking, GCS score, Braden score, and nasogastric tube were found to be important influencing factors for the occurrence of SAP in older adult patients with hemorrhagic stroke.

#### 4.2.1 The impact of age on SAP in older adult patients with hemorrhagic cerebral stroke and its nursing interventions

In this study, each additional year of age was associated with an ~6.4% increase in the odds of developing SAP. Consistent with previous studies (25, 26), older adult stroke patients experience gradual decline in immune function and resistance, leading to reduced stress tolerance and increased susceptibility to SAP. Additionally, Assefa et al. (27) demonstrated that stroke patients aged ≥75 years had a 4-fold higher risk of developing SAP compared to those aged 18–44 years. With socioeconomic development, China's aging rate is projected to reach 38.1% by 2050 (28), suggesting a potential further increase in SAP prevalence among older adult populations. Therefore, interventions to enhance immune function and resistance in older adult hemorrhagic stroke patients are particularly crucial. In clinical nursing practice, strengthening nutritional support to ensure adequate nutrient intake can help improve immune system function. Additionally, encouraging patients to engage in appropriate active exercise or providing assisted passive movement by nursing staff may improve physical constitution and disease resistance. Health education for patients and their families regarding hemorrhagic stroke and SAP is also essential to enhance self-management capacity and reduce complications. Facing the challenges posed by population aging, nursing practice should prioritize effective management and intervention for older adult hemorrhagic stroke patients to reduce SAP incidence and ultimately improve quality of life (11).

#### 4.2.2 Impact of smoking history on the risk of SAP in older adult hemorrhagic stroke patients and nursing interventions

The results of this study showed that the probability of older adult hemorrhagic stroke patients with a smoking history developing stroke-associated pneumonia (SAP) was 2.488-fold that of patients without a smoking history. In contrast, in a previous study (29), the impact of smoking on the risk of SAP was 1.12-fold. Additionally, nicotine, as the main component of tobacco, has been proven to damage the respiratory mucosa and affect the ability of the respiratory tract to clear secretions (30, 31). This makes it easier for sputum to accumulate in the lungs, thereby increasing the risk of pulmonary inflammation. This indicates that a smoking history significantly increases the risk of SAP in older adult hemorrhagic stroke patients. Therefore, it is particularly important to strengthen respiratory tract nursing for patients with a smoking history. Specific measures include regularly clearing respiratory tract secretions and assisting with sputum excretion to reduce the accumulation of sputum in the lungs and lower the possibility of pulmonary infection. At the same time, regularly monitor the patients' pulmonary status to detect and address potential problems in a timely manner, thus effectively reducing and delaying the risk of SAP in older adult hemorrhagic stroke patients and improving their rehabilitation quality.

#### 4.2.3 Relationship between GCS score and the risk of SAP in older adult hemorrhagic stroke patients and nursing interventions

The results of this study indicated that the Glasgow Coma Scale (GCS) score of older adult hemorrhagic stroke patients was negatively correlated with the risk of SAP, which was similar to a previous study (6). This may be related to the fact that the hematoma compresses the surrounding blood vessels and nerve tissues, the respiratory center is inhibited, and the patients experience impaired consciousness. As a result, they may not be able to cough effectively and clear respiratory tract secretions, and the gas-exchange function of the lung tissue is significantly insufficient, which in turn increases the possibility of SAP (20, 32). Based on the above situation, for patients with a low GCS score, nursing interventions should focus on the following aspects: First, maintaining a patent airway is fundamental. If necessary, auxiliary breathing equipment such as mechanical ventilation can be used to improve the gas-exchange function of the lungs. Second, reasonable position management is also crucial. It is recommended that patients take a lateral or prone position to promote the drainage of respiratory tract secretions and reduce the risk of aspiration. In addition, when the patients' condition allows, rehabilitation training should be carried out as early as possible to promote the recovery of consciousness and the improvement of respiratory function. Active management of lung function is also an important measure to prevent complications such as SAP. Finally, psychological nursing and family education should not be ignored. The psychological state of the patients or their families may affect the treatment effect. Therefore, providing psychological support and health education can help improve treatment compliance.

#### 4.2.4 Relationship between Braden score and the risk of SAP in older adult hemorrhagic stroke patients and nursing interventions

This study showed that the lower the Braden score of older adult hemorrhagic stroke patients, the higher the risk of SAP, which was consistent with the study by Ding et al. (33). The Braden score includes six aspects: sensory perception, moisture, activity, nutrition, mobility, friction, and shear force. Older adult hemorrhagic stroke patients often have reduced or lost sensory perception due to nerve function impairment and are unable to perceive skin discomfort or pain. In addition, due to limited mobility, patients are prone to sweating or having excrement residues, which makes the skin moist, weakens its barrier function, and increases the risk of damage and infection. At the same time, due to nerve function impairment or muscle weakness, patients have difficulty in self-activity or changing positions and are prone to long-term bed rest, which increases the risk of SAP. Older adult hemorrhagic stroke patients may also be temporarily prohibited from eating due to the condition, resulting in digestive and absorption dysfunction and malnutrition, which weakens the immune function and further increases the occurrence of SAP. Overall, a lower Braden score means that patients may have more severe nerve function impairment, weaker activity ability, and poorer nutritional status at admission, reflecting situations such as long-term bed rest, malnutrition, and limited activity. These factors can lead to a decline in immune function and damage to skin integrity, increasing the possibility of SAP (33). Therefore, medical staff need to comprehensively understand the patients' health status and nursing needs and formulate personalized preventive measures. For example, strengthen skin care and regularly check the pressure-bearing areas; provide appropriate nutritional support, monitor the patients' nutritional status through diet assessment and laboratory indicators (such as serum albumin and pre-albumin), and for patients who cannot eat normally, use enteral or parenteral nutrition to supplement necessary nutrients; at the same time, when the condition allows, start passive and active exercises as early as possible to promote the recovery of muscle strength and prevent muscle atrophy and joint stiffness. Through these comprehensive nursing intervention measures, the risk of patients developing SAP can be effectively reduced.

#### 4.2.5 Relationship between nasogastric tube feeding therapy and the risk of SAP in older adult hemorrhagic stroke patients and nursing interventions

This study found that when older adult hemorrhagic stroke patients received nasogastric tube feeding therapy, the risk of developing SAP increased by about 1.761-fold. Multiple studies (3, 20, 27) have shown that hemorrhagic stroke patients are prone to symptoms such as increased intracranial pressure, reflux, and vomiting. During the indwelling of the gastric tube, the gastric volume can increase sharply, causing spastic contraction and gastric retention. After the reflux of gastroesophageal secretions, a bacterial biofilm is formed, which in turn triggers SAP. Some patients receive acid-suppression therapy to prevent stress-related gastrointestinal bleeding, which may lead to alkalization of gastric juice and bacterial proliferation, thereby increasing the risk of SAP (1, 20). Therefore, for older adult hemorrhagic stroke patients, medical staff can weigh the treatment needs and potential complication risks when deciding whether to perform nasogastric tube feeding therapy. For patients who have already received nasogastric tube feeding therapy, timely assess whether the therapy still needs to be continued according to their recovery situation to avoid unnecessary risks. During nasogastric tube feeding therapy, elevating the patient's head position can reduce the chance of gastroesophageal reflux; suctioning oral sputum or secretions can reduce the risk of bacterial colonization and infection; providing easily digestible and nutritionally balanced nasogastric tube feeding fluid, avoiding high-fat and high-protein diets, can reduce the gastrointestinal burden; intermittently monitoring the gastric residual volume helps to understand the gastric emptying situation, preventing gastric retention and reflux. At the same time, use acid-suppression drugs reasonably under the guidance of doctors to avoid excessive alkalization of gastric juice and prevent excessive bacterial proliferation, thereby reducing the risk of SAP and improving the safety and comfort of treatment (1, 20, 34).

### 4.3 Comparison with other SAP prediction models

Smith et al. (5) constructed a SAP prediction model (the ISAN score) based on a large amount of data from patients with ischemic and hemorrhagic stroke collected through the Sentinel Stroke National Audit Programme (SSNAP). They also validated the application of the A^2^DS^2^ score. It was found that both the ISAN score (scoring items: age, gender, National Institutes of Health Stroke Scale (NIHSS), mRS) and the A^2^DS^2^ score had good predictive performance for patients with ischemic stroke, but poor performance for those with hemorrhagic stroke. Their C-statistics and 95% CI were 0.71 (0.66–0.77) and 0.72 (0.67–0.77), respectively, which were lower than the AUC of the LR model in the three datasets in this study (0.883, 0.855, and 0.882). Moreover, there were no records in SSNAP regarding factors such as patients' medication use and invasive treatment, which may affect the occurrence of SAP. Patients with hemorrhagic stroke often experience a sharp increase in intracranial pressure due to hematoma occupation, requiring more invasive procedures. These procedures can damage the normal physiological barriers of the human body, increasing the chance of bacterial infection and significantly raising the risk of SAP. In addition, the use of drugs may affect the immune status or bleeding risk, thus indirectly influencing the occurrence of SAP and reducing the predictive performance of the model for SAP in patients with hemorrhagic stroke. Ge et al. (10) used machine-learning methods such as Logistic regression, SVM, and deep neural networks to construct a risk prediction model for SAP in patients with acute ischemic stroke. The results showed that the deep neural network model had the highest AUC value, reaching 0.96, indicating better predictive efficacy than other machine-learning methods. However, it requires a large amount of data and complex calculations. The final prediction model included 25 factors, covering various aspects such as patients' basic information, vital signs, and laboratory test results. In clinical practice, obtaining such comprehensive and detailed data is time-consuming and labor-intensive. In some primary healthcare institutions or emergency situations, it may not be possible to obtain some of the data in a timely manner, which greatly limits the clinical application of this model. Furthermore, this model was developed based only on single-center data and has not undergone external validation. Its predictive performance and extrapolation ability in different regions and populations need further investigation. Yu et al. (4) established a logistic regression (LR) model for 378 patients with spontaneous intracerebral hemorrhage, which included IVH (IVH, intraventricular hemorrhage), hypertension, dysphagia, GCS, NIHSS, SIRI (the systemic inflammation response index), and PLR (platelet/lymphocyte ratio). However, this study excluded patients with infratentorial hemorrhage. Since infratentorial hemorrhage accounts for a certain proportion of intracerebral hemorrhage patients, excluding these patients may affect the accuracy and universality of the model. Meanwhile, the study did not clearly distinguish older adult patients. Older adult patients are at a higher risk of developing SAP due to factors such as decreased physical function and reduced immune function, and their disease progression and treatment responses may differ from those of younger patients. Additionally, this model relies more on laboratory indicators such as SIRI and PLR, which can be affected by various factors such as patients' underlying diseases, infection status, and treatment measures, limiting their stability and reliability. Moreover, there were also no records of patients' medication use and invasive treatment in the data collection. This was a single-center retrospective study, and external validation and multi-center studies are needed to further confirm the effectiveness and reliability of the model. Wang et al. (3) conducted a logistic regression analysis on 328 patients with acute stroke and found that intracerebral hemorrhage, indwelling nasogastric tube, and high NLR (neutrophil/lymphocyte ratio) were predictors of SAP in these patients. However, this study included patients with underlying pulmonary diseases (including chronic bronchitis, asthma, and bronchiectasis). As an inflammatory indicator, NLR has a relatively high baseline level in patients with underlying pulmonary diseases because these diseases themselves can lead to a chronic inflammatory state. Including these patients may confound the association between NLR and SAP and affect the predictive performance of the model. Therefore, this study focused on constructing a dedicated prediction tool for the high-risk group of older adult patients with hemorrhagic stroke. The prediction factors of this tool include age, smoking, GCS, Braden scale score, and indwelling nasogastric tube. These indicators can be quickly evaluated at the bedside and have good clinical practicability. By collecting multi-center data, internal and external validation confirmed the generalization ability of the model, and a nomogram visualization tool was constructed, making it possible to perform rapid bedside risk assessment.

## 5 Limitation

This study has certain limitations. Firstly, regarding the study subjects, the sample size of this study mainly comes from tertiary medical centers in Guiyang, Guizhou Province, China. Although the multi-center design has improved the representativeness of the sample to some extent, China has a vast territory and there are differences in the allocation of medical resources among different regions. This leads to limited applicability of the model in other regions. In essence, this regional difference objectively reflects the heterogeneity of real-world data. Additionally, the unique altitude environment in Guizhou has formed hemodynamic characteristics, making the population in this area special. The excellent performance of the model in this population provides a differentiated baseline for subsequent validation in plain areas. Secondly, although the dataset is multi-center, it consists of retrospective data, which may not fully reflect current medical practices. Meanwhile, due to the lack of long-term follow-up data, it is impossible to evaluate the quality of life of patients after discharge. Future studies need to focus on clarifying these relationships.

## 6 Conclusion

In conclusion, this study focused on the construction and validation of a SAP risk prediction model for older adult patients with hemorrhagic stroke. The research found that among the four machine learning algorithms (XGBoost, LR, SVM, and Naive Bayes), the LR model demonstrated superior and more stable performance in predicting SAP in older adult patients with hemorrhagic stroke. Through internal and external validations using multi-center data, it was confirmed that this model has good predictive efficacy and generalization ability. The research results indicate that age, smoking, low GCS score, low Braden score, and the use of nasogastric tube are important influencing factors for SAP in older adult patients with hemorrhagic stroke. These indicators are all easily obtainable in clinical settings and facilitate rapid bedside assessment and application, and have good clinical operability. Compared with existing literature, the multi-center data in this study enhanced sample representativeness, the internal and external validations achieved optimization and ensured the predictive performance of the model, and a nomogram visualization tool was developed to achieve rapid bedside risk assessment. This provides scientific evidence for clinical medical staff, helps achieve early identification of SAP in older adult patients with hemorrhagic stroke, and through strengthening nutritional support, respiratory management, postural care, skin care, and reasonable nasogastric feeding and other comprehensive nursing intervention measures, reduces or delays the occurrence of SAP and improves patient prognosis, providing an operational basis for clinical nursing practice and prevention strategies.

## Data Availability

The original contributions presented in the study are included in the article/Supplementary material, further inquiries can be directed to the corresponding authors.
