# Supplementary material for: Development and validation of a machine learning-based risk prediction model for stroke-associated pneumonia in older adult hemorrhagic stroke
Source: Front Neurol. 2025 Jun 18;16:1591570. doi: 10.3389/fneur.2025.1591570 (PMC12214254; doi:10.3389/fneur.2025.1591570)
Supplement: Supplementary file 2 [file Data_Sheet_2.docx]

Supplementary Material

# 1 Supplementary Tables

**Supplementary Table 1.** Performance of Four Models in the Training Set

| Variables | tolerance | VIF |
| --- | --- | --- |
| Age | 0.851 | 1.176 |
| Smoking | 0.866 | 1.155 |
| GCS | 0.780 | 1.281 |
| mRS | 0.786 | 1.271 |
| Braden | 0.728 | 1.374 |
| SBP | 0.446 | 2.243 |
| DBP | 0.429 | 2.329 |
| WBC | 0.156 | 6.392 |
| Neut | 0.134 | 7.450 |
| Lym | 0.544 | 1.839 |
| Glu | 0.959 | 1.042 |
| Use of acid suppressants | 0.843 | 1.186 |
| Nasogastric tube | 0.907 | 1.103 |

**Supplementary Table 2.** Performance of Four Models in the Training Set

|  | auc_ci | sens_ci | spes_ci | accuracy_ci | precision_ci | recall_ci | f1_score_ci |
| --- | --- | --- | --- | --- | --- | --- | --- |
| XGBoost | 0.883 (0.853-0.913) | 0.710 (0.650 - 0.771) | 0.883 (0.843 - 0.923) | 0.803 (0.764-0.838) | 0.840 (0.786 - 0.893) | 0.710 (0.650 - 0.771) | 0.770 (0.786 - 0.893) |
| Logistic Regression | 0.883 (0.853-0.912) | 0.766 (0.710 - 0.823) | 0.839 (0.793 - 0.884) | 0.805 (0.766-0.840) | 0.804 (0.749 - 0.858) | 0.766 (0.710 - 0.823) | 0.785 (0.749 - 0.858) |
| SVM | 0.889 (0.860-0.918) | 0.883 (0.840 - 0.926) | 0.714 (0.657 - 0.770) | 0.792 (0.752-0.828) | 0.727 (0.673 - 0.781) | 0.883 (0.840 - 0.926) | 0.797 (0.673 - 0.781) |
| Naive Bayes | 0.865 (0.832-0.898) | 0.808 (0.756 - .861) | 0.778 (0.727 - 0.830) | 0.792 (0.752-0.828) | 0.759 (0.703 - 0.814) | 0.808 (0.756 - 0.861) | 0.783 (0.703 - 0.814) |

**Supplementary Table 3.** Performance of Four Models in the Internal Validation Set

|  | auc_ci | accuracy_ci | sens_ci | spes_ci | precision_ci | recall_ci | f1_score_ci |
| --- | --- | --- | --- | --- | --- | --- | --- |
| XGBoost | 0.850 (0.796-0.904) | 0.796 (0.733-0.850) | 0.695 (0.602 - 0.787) | 0.891 (0.830 - 0.952) | 0.857 (0.779 - 0.935) | 0.695 (0.602 - 0.787) | 0.767 (0.779 - 0.935) |
| Logistic Regression | 0.855 (0.803-0.907) | 0.791 (0.727-0.846) | 0.747 (0.660 - 0.835) | 0.832 (0.759 - 0.905) | 0.807 (0.724 - 0.889) | 0.747 (0.660 - 0.835) | 0.776 (0.724 - 0.889) |
| SVM | 0.835 (0.779-0.892) | 0.796 (0.733-0.850) | 0.674 (0.579 - 0.768) | 0.911 (0.855 - 0.966) | 0.877 (0.801 - 0.952) | 0.674 (0.579 - 0.768) | 0.762 (0.801 - 0.952) |
| Naive Bayes | 0.836 (0.779-0.893) | 0.806 (0.744-0.859) | 0.737 (0.648 - 0.825) | 0.871 (0.806 - 0.937) | 0.843 (0.765 - 0.922) | 0.737 (0.648 - 0.825) | 0.787 (0.765 - 0.922) |

# 2 Supplementary Figures


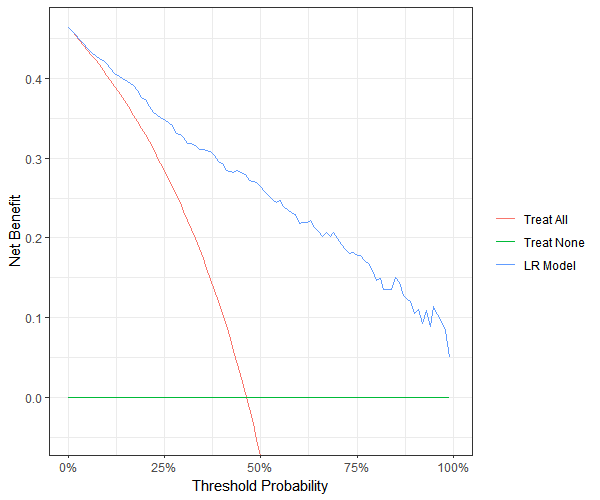


**Supplementary Figure 1.** The red diagonal line represents the intervention for all patients regardless of cost, the horizontal green line represents no intervention for all patients regardless of consequences, and the blue line represents the standardized net benefit of this model.
